# Functional and molecular characterization of cancer stem-like cells in bladder cancer: a potential signature for muscle-invasive tumors

**DOI:** 10.18632/oncotarget.5517

**Published:** 2015-10-05

**Authors:** Margarida Ferreira-Teixeira, Belmiro Parada, Paulo Rodrigues-Santos, Vera Alves, José S. Ramalho, Francisco Caramelo, Vitor Sousa, Flávio Reis, Célia M. Gomes

**Affiliations:** ^1^ Laboratory of Pharmacology and Experimental Therapeutics, Institute for Biomedical Imaging and Life Sciences (IBILI), Faculty of Medicine, University of Coimbra, Coimbra, Portugal; ^2^ CNC.IBILI, University of Coimbra, Coimbra, Portugal; ^3^ Urology and Renal Transplantation Department, Coimbra University Hospital Centre (CHUC), Coimbra, Portugal; ^4^ Immunology and Oncology Laboratory, Center for Neurosciences and Cell Biology (CNC), University of Coimbra, Coimbra, Portugal; ^5^ Institute of Immunology, Faculty of Medicine, University of Coimbra, Coimbra, Portugal; ^6^ Center of Investigation in Environment, Genetics and Oncobiology (CIMAGO), Faculty of Medicine, University of Coimbra, Coimbra, Portugal; ^7^ CEDOC, Faculty of Medical Sciences, New University of Lisbon, Lisbon, Portugal; ^8^ Laboratory of Biostatistics and Medical Informatics, Faculty of Medicine, University of Coimbra, Coimbra, Portugal; ^9^ Institute of Anatomical and Molecular Pathology, Faculty of Medicine, University of Coimbra, Coimbra, Portugal; ^10^ Service of Anatomical Pathology, Coimbra University Hospital Centre (CHUC), Coimbra, Portugal

**Keywords:** bladder cancer, cancer stem cells, ALDH, stemness markers, chemoresistance

## Abstract

Striking evidence associates cancer stem cells (CSCs) to the high recurrence rates and poor survival of patients with muscle-invasive bladder cancer (BC). However, the prognostic implication of those cells in risk stratification is not firmly established, mainly due to the functional and phenotypic heterogeneity of CSCs populations, as well as, to the conflicting data regarding their identification based on a single specific marker. This emphasizes the need to exploit putative CSC-related molecular markers with potential prognostic significance in BC patients.

This study aimed to isolate and characterize bladder CSCs making use of different functional and molecular approaches. The data obtained provide strong evidence that muscle-invasive BC is enriched with a heterogeneous stem-like population characterized by enhanced chemoresistance and tumor initiating properties, able to recapitulate the heterogeneity of the original tumor. Additionally, a logistic regression analysis identified a 2-gene stem-like signature (SOX2 and ALDH2) that allows a 93% accurate discrimination between non-muscle-invasive and invasive tumors.

Our findings suggest that a stemness-related gene signature, combined with a cluster of markers to more narrowly refine the CSC phenotype, could better identify BC patients that would benefit from a more aggressive therapeutic intervention targeting CSCs population.

## INTRODUCTION

Bladder cancer (BC) is the fourth most common carcinoma among men and the ninth among women in the Western world [1]. Two independent carcinogenic pathways are clearly described, the papillary non-muscle-invasive and the muscle-invasive [2, 3]; about 70% of newly diagnosed cases are non-muscle-invasive, characterized by grown of hyperplastic urothelial cells toward the bladder lumen [4]. Even though these low grade tumors can be effectively treated by transurethral resection followed by intravesical chemotherapy, often with a good prognosis, they have a high propensity for recurrence [4, 5]. In fact, about 15% of these papillary bladder tumors will progress to muscle-invasive and metastatic cancers, which require long-term medical care, inevitably associated with high personnel and socioeconomic costs [6].

To date, there are no reliable prognostic markers in clinical practice able to identify and predict the subset of patients with papillary tumors that will progress into more aggressive muscle-invasive forms. It is currently believed that the high frequency of recurrence in superficial papillary BC is related with the presence of a population of undifferentiated cells exhibiting stem-like properties, the so-called cancer stem cells (CSCs) or tumor initiating cells [7–9]. These undifferentiated cells are able to undergo unlimited self-renewal and are uniquely able to reform the tumors when implanted in immunocompromised mice [10, 11]. CSCs have been described in an increasing number of solid tumors and emerging evidences support that BC arises by transformation of basal layer urothelial cells, based on the expression of surface markers similar to that of normal basal cells [12–14]. The clinical implications of CSCs are attributed to the enhanced tumorigenic potential and ability to dictate invasion and metastatic progression and enhanced resistance therapy through a variety of mechanisms including quiescence or slow cycle kinetics, enhanced DNA repair mechanisms and overexpression of multidrug resistance-type membrane transporters, which overalls contribute to the failure of existing therapies [15–17].

Although in a clinical context the role of CSCs in BC has gained considerable support, conflicting data exist and the prognostic significance of these cells is still not firmly established. The main cause for this doubt is the lack of a universal CSC marker, together with variable levels of expression according to the tumor stage, as well as, the ultimate lack of correlation between the commonly tested CSCs surface markers and the patient outcome/resistance to therapy. Other confounding factors are the functional and phenotypic heterogeneity of CSCs and their plasticity in the context of tumor development and progression [18, 19]. Being BC a highly heterogeneous disease beyond the dual pathway carcinogenesis, it is likely that a manifest heterogeneity exists at the stem cell level that cannot be assessed by using a “one-marker” approach. For example, attempts to isolate bladder CSCs based on the basal cell surface marker CD44 expression showed substantial variation among basal tumor subtypes and have been unsuccessful in non-muscle-invasive tumors. Furthermore, sorted CD44^−^ cells were able to form tumors when engrafted in nude mice, which is a major hallmark of CSCs, reflecting both the ambiguity of CD44 in CSCs maintenance and the variable phenotypic profile within bladder CSCs populations [6, 20, 21]. Likewise, several groups have been proposing that high aldehyde dehydrogenase (ALDH) activity is an accurate predictive marker for the identification of CSCs in several types of tumors, including in BC [22–24]. However, conflicting studies reveal that ALDH may be highly expressed in tumorigenic cells that cannot be phenotypically characterized as CSCs [20]; in addition, it has been suggested that the presence of different ALDH isoforms might be the main responsible for tumor progression [25, 26].

In this study we performed a functional and molecular characterization of human bladder CSCs in human cell lines and primary clinical samples, using different functional approaches and a plethora of distinct stem cell-related markers, including embryonic transcription factors [OCT4 (POU5F1), SOX2 and NANOG], aldehyde dehydrogenase isoforms (ALDH1A1, ALDH2 and ALDH7A1), ABC transporters [PGP (ABCB1) and BCRP (ABCG2)] and basal urothelial stem cell markers (CD44, CD47 and KRT14). Our results provided evidence that the muscle-invasive BC harbor distinct cell subsets reflecting molecular features of stem-like cells, together with an aggressive phenotype characterized by enhanced chemoresistance and tumor initiating ability recapitulating the heterogeneity of the original tumor in a specific organ microenvironment. The gene expression pattern analysis in primary clinical samples allowed the identification of a two-gene stem-like signature (SOX2 and ALDH2) that could be useful to ascertain patients with muscle-invasive tumors that are more susceptible to disease progression or metastasis development.

## RESULTS

### BC cells contain sphere-forming cells expressing stemness-related markers

**Figure 1:**
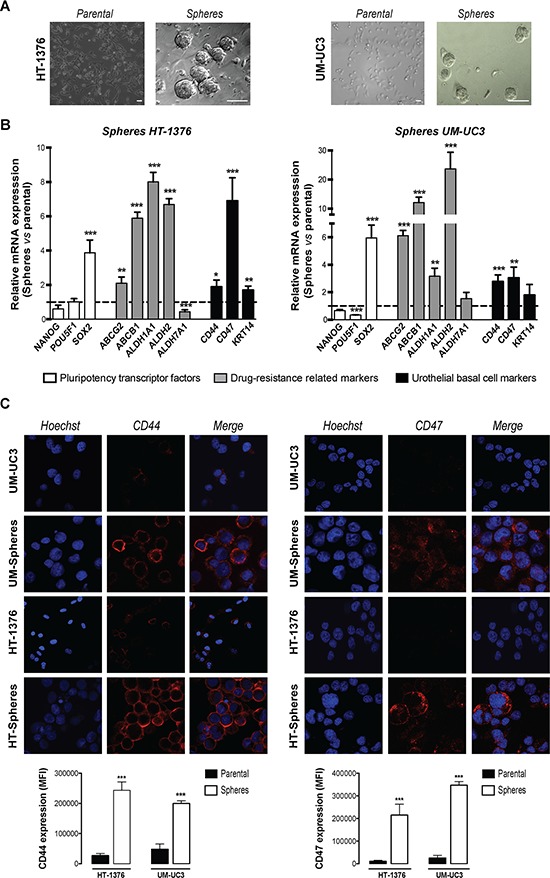
BC cells contain a population of sphere-forming cells with stem-like properties **A.** Parental monolayer HT-1376 and UM-UC3 cells form spherical colonies generated from a single cell, when cultured in serum-free DMEM/F12 medium, supplemented with bFGF, EGF and B27 supplement in Matrigel coated plates after 11 days. Scale bars = 50 μm. **B.** qRT-PCR analysis expression of pluripotency-related transcription factors (NANOG, POU5F1, SOX2), drug-resistance related genes (ABCG2, ABCB1, ALDH1A1, ALDH2, ALDH7A1) and urothelial basal cell-related markers (CD44, CD47 and KRT14) by qRT–PCR. The graph shows the fold change in gene expression in spheres relative to parental cell line that was set as 1 (mean + SEM, *n* = 4). **C.** Representative immunofluorescence staining of CD44 and CD47 monoclonal antibodies in spheres and corresponding parental cells. Cell nuclei were counterstained with Hoechst 33258 (in blue). The graph shows the mean fluorescence intensities in the confocal micrographs (*n* = 3). *p < 0.05, **p < 0.01, ***p < 0.001 compared to corresponding parental cells.

HT-1376 and UM-UC3 BC cell lines were allowed to growth in matrigel coated plates with serum-free DMEM/F12 medium supplemented with basic fibroblast growth factor (bFGF), human recombinant epidermal growth factor (EGF) and B27 to assess the presence of putative CSCs. Following a period of culture, cells formed visible spherical colony-like structures that continued to grow reaching 50 μm diameter at day 11 (Figure 1A). After plating in adherent conditions, these cells adhered to the bottom of the flask and acquired a similar morphology to their respective parental cells (data not shown), thus demonstrating their ability to generate differentiated progeny.

These cells were further characterized regarding expression of putative stemness-related markers by qRT-PCR, grouped in three categories: pluripotency transcription factors specific to embryonic stem cells (SOX2, POU5F1 and NANOG); urothelial basal cell-specific markers (CD44, CD47 and KRT14) and drug resistance-related genes including the ATP-binding cassette transporters (ABCG2 and ABCB1) and isoforms of ALDH (ALDH1A1, ALDH2 and ALDH7A1). The genes were selected based on their seeming functions in stem cells [27]. This analysis was conducted in both adherent and corresponding sphere-forming cells.

The mRNA expression levels of SOX2 were significantly up regulated (~4-fold, *p* < 0.001) in sphere-forming cells when compared to the parental cells. There was unchanged expression of the other transcription factors analyzed (POU5F1 and NANOG). The drug efflux transporters ABCG2 (*p* < 0.01) and ABCB1 (*p* < 0.001) and the ALDH1A1 and ALDH2 (*p* < 0.01) isoforms of ALDH, showed a consistent up-regulation in sphere-forming cells relatively to their parental counterparts (Figure 1B). No significant changes were observed on ALDH7A1 isoform between spheres and adherent cells. Regarding the expression of urothelial basal cell-related markers, both sphere-forming cells showed a significant mRNA up-regulation of CD44 (*p* < 0.05) and CD47 (*p* < 0.01), which were further confirmed at the protein level by immunofluorescence. In addition, sphere-derived cells showed a marked membranous immunoreactivity for CD44 and CD47, in opposition with the parental cells that showed nearly no detectable staining for both markers (Figure 1C). Although a higher expression of the basal keratin marker KRT14 was found by immunofluorescence staining in HT-1376-spheres, the differences did not achieved statistical significant when compared with the parental cells (data not shown).

### Sphere-forming cells are enriched in ALDH^+^ cells expressing stemness-related markers

**Figure 2:**
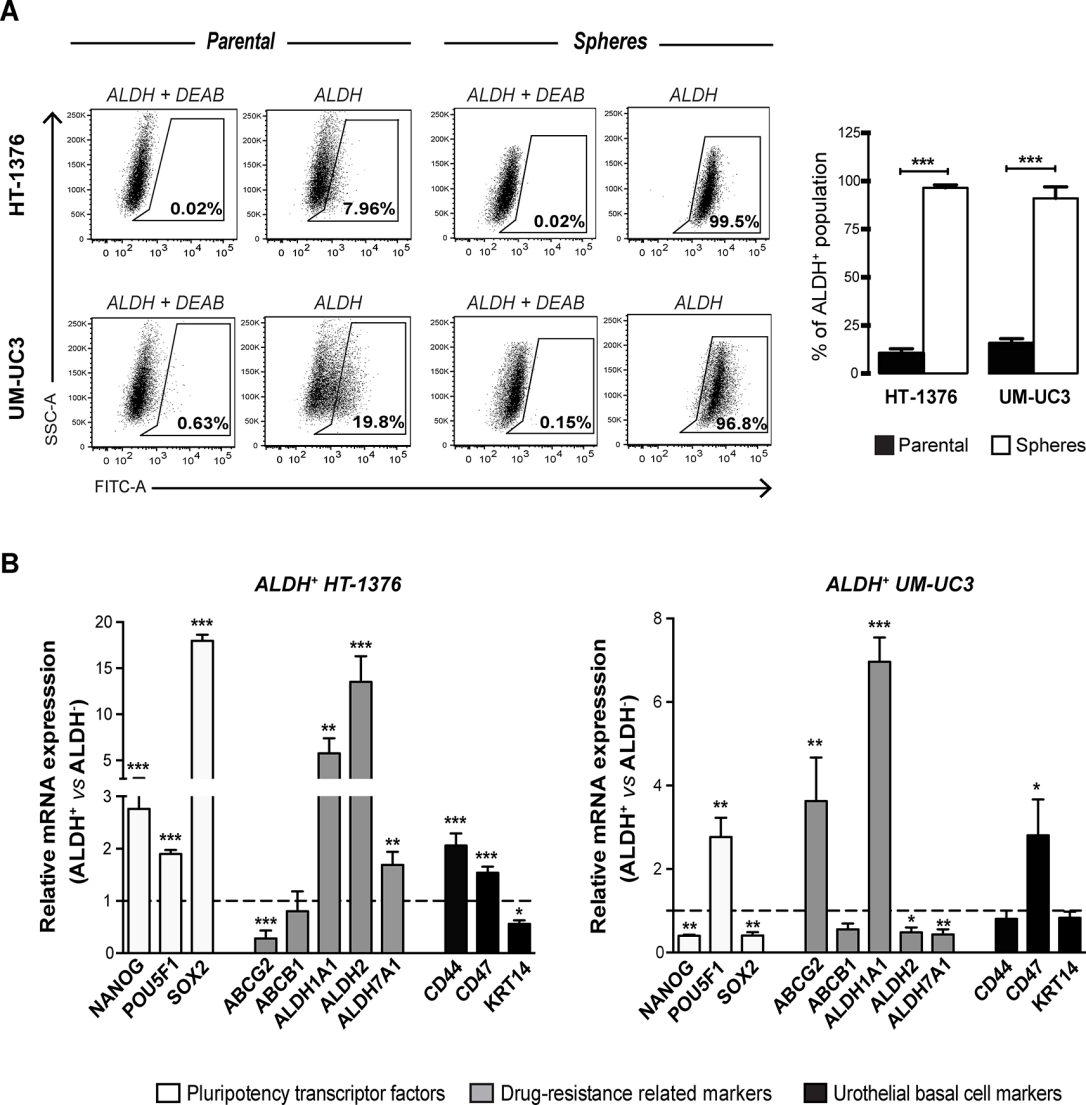
Sphere-forming cells are enriched in ALDH-positive cells expressing cancer stem-like markers **A.** Representative flow cytometry analysis of ALDH enzymatic activity in parental and corresponding sphere-forming cells, showing a tremendous enrichment of ALDH^+^ cells in spheres. The ALDH inhibitor DEAB was used as a negative control to establish the baseline fluorescence of the cells. Cells without inhibitor that shifted to the right were considered ALDH^+^ cells. Bar graph shows the percentage of ALDH^+^ cells in parental and sphere cells (mean + SEM, *n* = 3). **p < 0.01, ***p < 0.001 compared to control adherent cells. **B.** qRT-PCR analysis of mRNA expression of pluripotency-related transcription factors (NANOG, POU5F1, SOX2), drug resistance-related genes (ABCG2, ABCB1, ALDH1A1, ALDH2, ALDH7A1 and urothelial basal cell-related markers (CD44, CD47 and KRT14) in ALDH^+^ and ALDH^−^ FACS sorted cells. Gene expression levels in ALDH^+^ cells were normalized to the respective ALDH^−^ population that was set as 1 (mean + SEM, *n* = 4). *p < 0.05, **p < 0.01 and ***p < 0.001 compared to corresponding ALDH^−^ cells.

In addition to the matrigel clonogenic assay, we also evaluated ALDH activity as a functional marker for identifying stem-like cell populations. Flow cytometry analysis revealed that both BC cell lines contain a percentage of cells displaying high ALDH activity (UM-UC3: 15.67 ± 2.49%; HT-1376: 10.50 ± 2.39%) as depicted in Figure 2A. To verify whether sphere-forming cells are enriched with ALDH^+^ cells we performed a flow cytometry analysis of ALDH activity in sphere-forming cells. The results showed that spheres isolated from either UM-UC3 or HT-1376 cell lines are highly enriched in ALDH^+^ cells, with percentages of 91.00 ± 6.00% (*p* < 0.001) and 96.5 ± 1.50% (*p* < 0.001), respectively (Figure 2A).

Subsequently, we examined the expression profile of stem cell-related genes in sorted ALDH^+^ and ALDH^−^ populations for comparison with spheres (Figure 2B). The ALDH^+^ fraction sorted from the HT-1376 cell line showed high expression levels of NANOG, POU5F1, SOX2, ALDH1A1, ALDH2, ALDH7A1, CD44 and CD47 (*p* < 0.01), when compared with the ALDH^−^ population. All these genes, with the exception of NANOG, POU5F1 and ALDH7A1, are commonly overexpressed in spheres and ALDH^+^ cells. The ALDH^+^ fraction of the UM-UC3 cell line exhibited overexpression of POU5F1, ABCG2, ALDH1A1 and CD47 (*p* < 0.05), sharing the last three with the sphere-derived cells.

Despite the similarities in gene expression between spheres and ALDH^+^ cells, the partial overlapping suggests the co-existence of distinct stem-like cells populations within BC. Thus, each functional assay we used appears to identify a unique subset of CSCs, rather than the same population of cells.

### Bladder cancer sphere-forming cells display increased resistance to cisplatin and methotrexate

**Figure 3:**
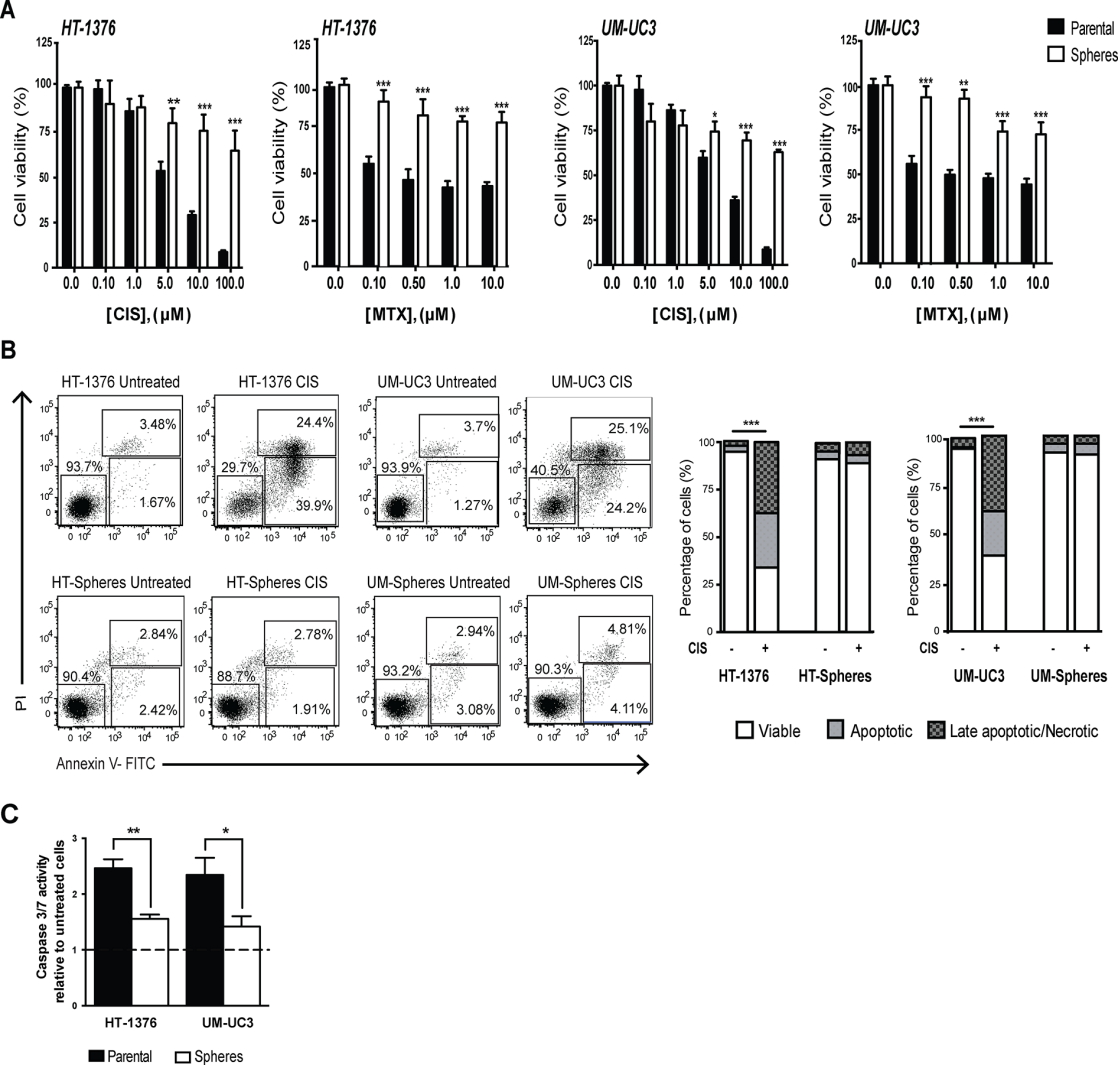
Sphere-forming cells display enhanced chemoresistance to CIS and MTX **A.** Cytotoxic effects of CIS and MTX in viability of parental BC cell lines (black bars) and corresponding sphere-forming cells (white bars) determined by the MTT assay. The percentage of viable cells was normalized to respective untreated control cells. **B.** Annexin V/PI flow cytometry analysis of apoptosis after treatment with 10 μM CIS for 48 h. The lower right quadrant (Annexin V-FITC^+^/PI^−^) was considered as early-stage apoptotic cells, the upper right quadrant (Annexin V-FITC^+^/PI^+^) was considered late-stage apoptotic cells, the upper left quadrant (Annexin V-FITC^−^/PI^+^) was considered as necrotic cell and the lower left quadrant (Annexin V-FITC^−^/PI^−^) was considered as viable cells. Bar graph represents the mean percentage of viable, early and apoptotic cells within each cell population (*n* = 3) ***p < 0.001 treated viable cells *vs.* untreated viable cells. **C.** Caspase-3/7 activity measured using the Caspase-Glo assay following cells treatment with 10 μM CIS for 48 h. Values were normalized to respective untreated control cells (mean + SEM, *n* = 3). *p < 0.05 and **p < 0.01 *vs.* treated parental cells.

To determine whether sphere-forming cells possess a chemoresistance phenotype, both cell populations were assayed for sensitivity to cisplatin (CIS) and methotrexate (MTX), two drugs currently used in the treatment of invasive BC. Both drugs induced a pronounced decrease in cell viability in a dose-dependent manner in parental cells, and had only a modest effect on the viability of corresponding sphere-forming cells, as assessed by the MTT assay (Figure 3A). The same chemoresistance profile was observed in spheres that were previously dissociated and cultured in monolayer, suggesting that the culture model (2D monolayer *versus* 3D spheres) does not influence the drug bioavailability and cells drug susceptibility (data not shown).

We also analyzed cells’ susceptibility to CIS-inducing apoptosis by measuring Annexin V staining by flow cytometry analysis and caspase 3/7 activity. This study was only performed for CIS, since MTX is a cytostatic agent that does not directly induces apoptosis.

As shown in Figure 3B, treatment with 10 μM CIS failed to induce apoptosis in sphere-forming cells. The percentage of apoptotic cells was very low, to levels similar to those of basal untreated controls; in contrast, a significantly higher percentage of early and late apoptotic cells was obtained in the parental cells under the same treatment conditions (*p* < 0.001). Likewise, caspase 3/7 activity, measured using the caspase-Glo assay, was significantly lower (*p* < 0.05) in spheres, compared to parental cells upon 48 h treatment with CIS (Figure 3C). Taken together, these results indicate that sphere-forming cells display a clear chemoresistance profile to the chemotherapeutic agents currently used in clinical practice.

### Enrichment of bladder cancer stem-like cells following short-term chemotherapy

**Figure 4:**
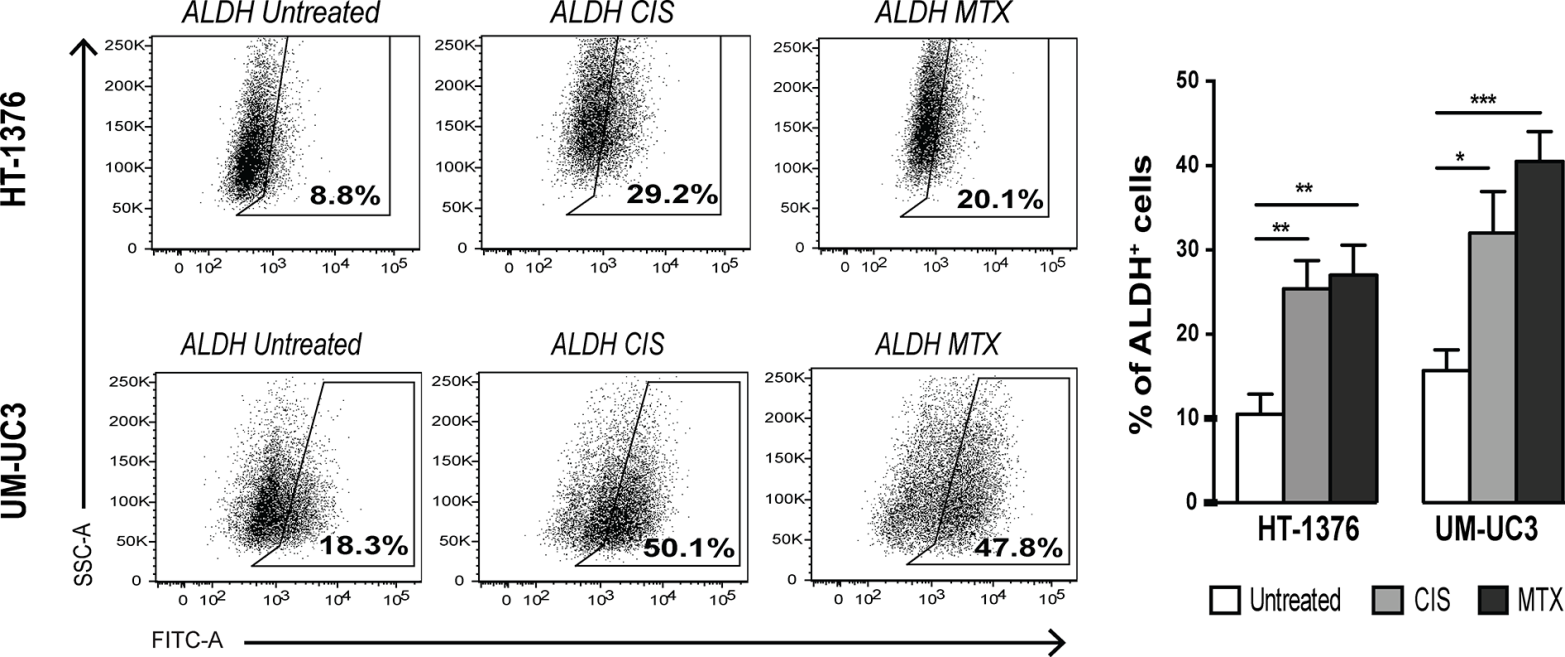
Short-term chemotherapy treatment induces enrichment of ALDH^+^ cells in BC cell lines Representative FACS dot-plots of ALDH activity in HT-1376 or UM-UC3 cells after treatment with 10 μM CIS or 10 μM MTX during 24 h and quantification of the percentage of ALDH^+^ cells (mean + SEM, *n* = 3). *p < 0.05, **p < 0.01 and ***p < 0.001 compared to untreated cells.

After demonstrating that sphere-derived cells are highly resistant to MTX and CIS, we tested whether short-term exposure to these drugs might induce stem cell-like properties in parental cells, using ALDH activity as readout of stemness, since this enzyme plays a vital role in cellular detoxification. Flow cytometry analysis showed a significant increase in the percentage of ALDH^+^ cells from 10.50 ± 2.39% to 25.40 ± 3.33% (*p* < 0.01) in HT-1376 cells and from 15.67 ± 2.49% to 32.00 ± 4.93% (*p* < 0.05) in UM-UC3 cells after 24 h of treatment with 10 μM CIS (Figure 4). MTX promoted similar effects in both cell lines, increasing the percentage of ALDH^+^ cells from 10.50 ± 2.39% to 27.00 ± 3.56% (*p* < 0.01) and from 15.67 ± 2.49% to 40.50 ± 3.52% (*p* < 0.001) in HT-1376 and UM-UC3, respectively. This increase in ALDH^+^ population seemingly to be due mainly to a phenotypic transition and not to the selection of ALDH^+^ cells that survive to therapy, since we did not observe a substantial increase in the percentage of dead cells after exposure to chemotherapeutic agents (see Supplementary Figure S1). This finding suggests that a short-term single exposure to chemotherapy induces a specific phenotypic transition to a chemo-tolerant state that offers an initial survival advantage against anti-cancer treatments.

### Bladder CSCs display high tumorigenic potential *in vivo* and generate a tumor resembling the patterns of a primary tumor

**Figure 5:**
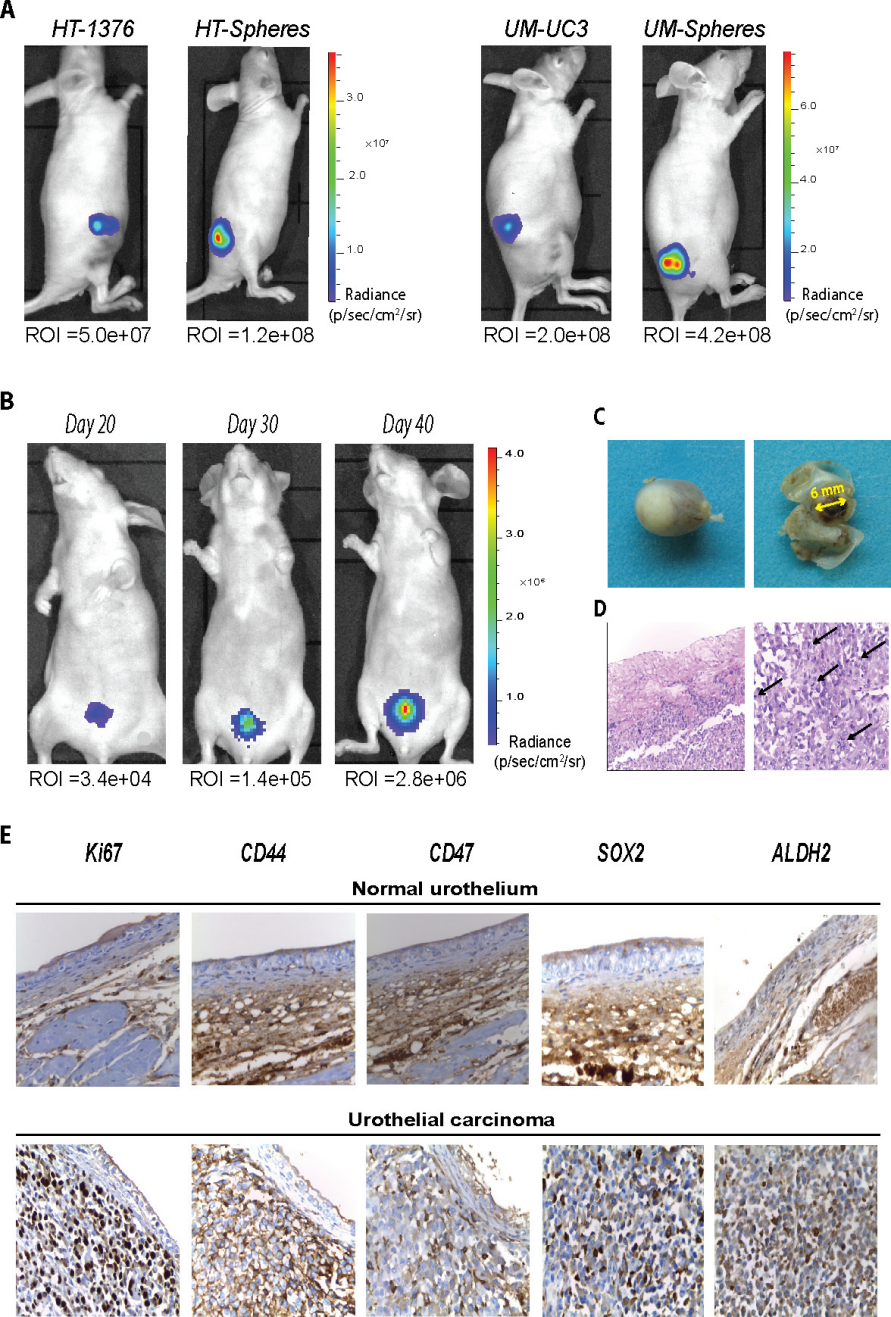
Sphere-forming cells present high tumorigenic potential and reproduce an orthotopic tumor resembling the clinical features of muscle-invasive BC **A.** Representative BLI images of mice inoculated subcutaneously with 1×10^4^ of parental or corresponding sphere-forming cells on day 30 post-inoculation. The tumor size was evaluated by quantification of bioluminescent signal (photons/sec/cm^2^/sr) in a region of interest (ROI) drawn around the tumor. **B.** Representative BLI images of a mouse inoculated intravesically with sphere-forming HT-1376 cells over 5 weeks. **C.** Macroscopic examination of a papillary tumor protruding into the bladder lumen on day 40. **D.** Representative H&E–stained section with invasion into the muscular layer (x200) and high mitotic count (x400). **E.** Immunohistochemical staining for Ki67, CD44, CD47, SOX2 and ALDH2 in serial sections of normal urothelium (upper row) and in the orthotopic tumor (lower row). Original magnification: x400.

Since the tumorigenic potential is considered a hallmark of CSCs, we compared the ability of spheres and respective parental cells to form subcutaneous tumors in nude mice. Both cell populations resulted in the generation of visible tumors; however, spheres were able to form larger tumors when compared with the corresponding parental cells, as indicated by the over 2-fold higher bioluminescent signal intensity observed in both the HT-1376 and UM-UC3 cell lines (Figure 5A). After confirming the enhanced tumorigenic potential of CSCs, we evaluated the ability of these cells to generate a primary tumor resembling the clinical condition of invasive urothelial carcinoma by orthotopic implantation. For that we inoculated by intravesical instillation HT-1376-derived spheres and respective parental cells into the bladder of nude mice. Ten days post-implantation, a bioluminescent signal was visible in the bladder that grew exponentially along 5 weeks (Figure 5B). At that moment, the animals showed sporadic hematuria, which is the most widely presented clinical sign of BC and were euthanized. Necropsy examination showed a solitary papillary tumor located on the posterior right bladder wall protruding into the bladder lumen (Figure 5C). None developed distant metastasis or tumor obstruction of the urethra. H&E staining revealed the presence of large neoplastic cells with high cellular and nuclear pleomorphism, large nuclei and high mitotic count with atypical mitosis, and with some infiltrating the muscular layer, resembling the histopathological features of a typical high grade muscular-invasive bladder carcinoma (Figure 5D). Immunohistochemical analysis revealed high levels of proliferating cells characterized by robust nuclear Ki67 staining and cytoplasmic CD44 and ALDH2 reactivity. The cell-surface protein CD47, together with CD44 and ALDH2, that were found up regulated at gene level in the CSCs subset, were also overexpressed in a high fraction of tumor cells (Figure 5E). Consistent with previous gene expression results, SOX2, the key gene regulator of pluripotency, was detectable in about 30% of the urothelial carcinoma, thus emphasizing the presence of different stem-like and differentiated cell populations within the bladder carcinoma. Indeed, the expression of those markers within the normal urothelium was negative/weak for Ki67 and low for CD44, CD47, ALDH2 and SOX2.

### Muscle-invasive BC samples overexpress SOX2, ALDH1A1 and ALDH2

**Figure 6:**
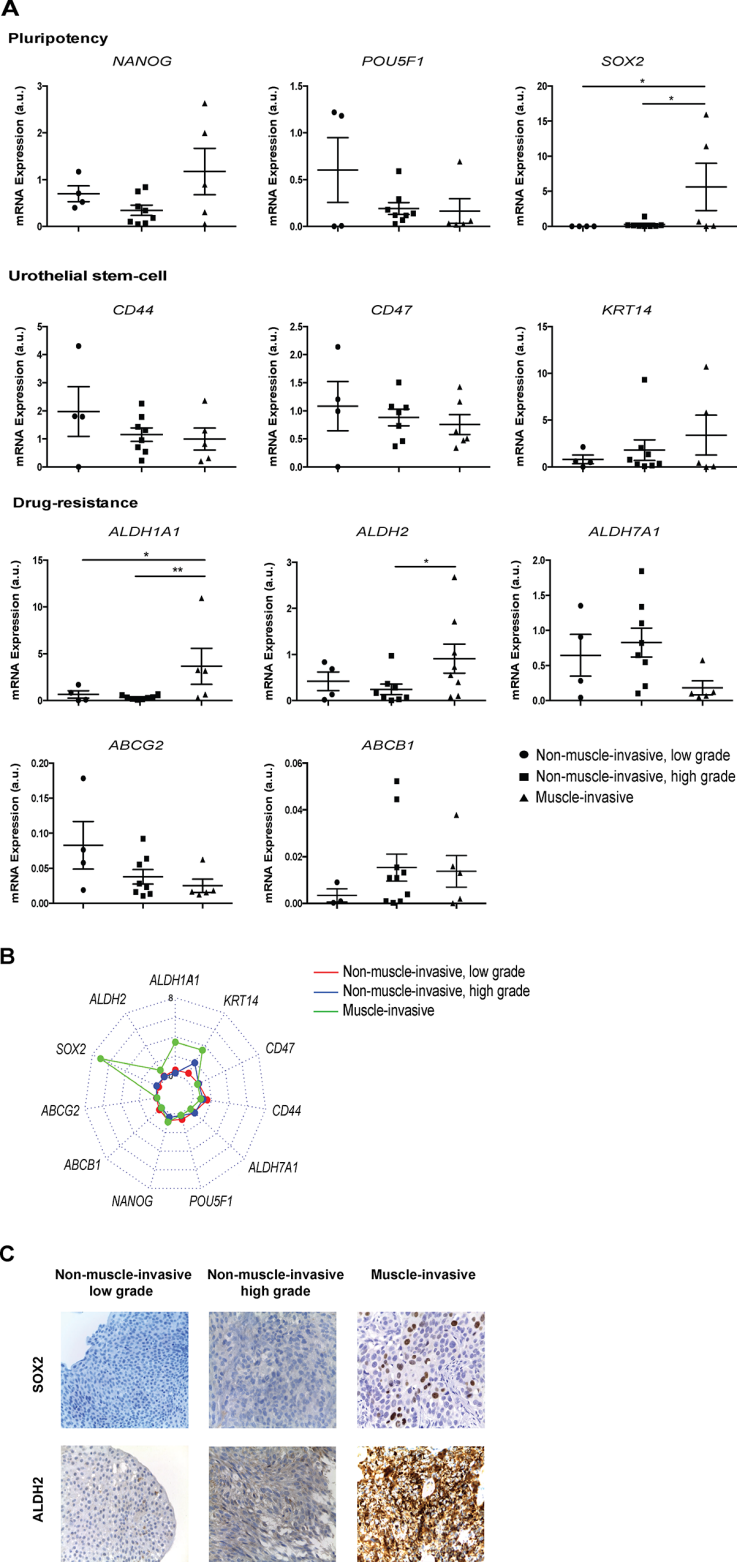
A two-gene stemness signature, SOX2 and ALDH2, discriminates muscle-invasive from non-muscle-invasive BC **A.** qRT-PCR analysis of mRNA expression of pluripotency-related transcription factors (NANOG, POU5F1, SOX2), drug resistance-related genes (ABCG2, ABCB1, ALDH1A1, ALDH2, ALDH7A1) and urothelial basal cell-related markers (CD44, CD47 and KRT14) in BC samples divided in three groups based on tumor grade: non-muscle-invasive, low grade; non-muscle-invasive, high grade and muscle-invasive. The expression levels of target genes in each sample were normalized to three housekeeping genes and are shown in scatter plots *p < 0.05, **p < 0.01 compared with low grade or high grade non-muscle-invasive tumors. **B.** Radar chart of all genes expression in non-muscle-invasive, low and high grade (red and blue lines, respectively) and muscle-invasive (green line) tumors. The graphic only depict the mean value for each gene. **C.** Immunohistochemical staining for SOX2 (upper row) and ALDH2 (lower row) in serial sections of tumoral tissue from BC patients with non-muscle-invasive (low grade), non-muscle-invasive (high grade) and muscle-invasive tumors. Original magnification: x400.

To further verify whether differential expression of stem cell-like gene expression could discriminate between grade/stage of the disease, we examined the expression profile of the stem-cell related markers, previously analyzed in sphere-forming cells, in a panel of 17 BC samples classified based on pathology reports as non-muscle-invasive low grade (*n* = 4), non-muscle-invasive high grade (*n* = 8) and muscle-invasive (*n* = 5). The expression of target genes was normalized to three internal reference genes. All genes were expressed at variable levels among the different samples analyzed (Figure 6A). The expression of the pluripotency transcription factor SOX2 and of ALDH1A1 and ALDH2 isoforms, were significantly increased in muscle-invasive tumors when compared to the non-muscle-invasive groups (*p* < 0.05). Conversely, the surface markers CD44 and CD47, together with the ABC transporters, the transcription factor POU5F1 and the ALDH7A1 isoform were almost equally distributed in the different BC samples and do not provide discriminatory information regarding grading stages of the disease. Although statistically not significant, the results showed a clear trend towards an up regulation of NANOG and KRT14 in muscle-invasive tumors. A radar chart of the mean values of all genes expression in non-muscle-invasive, low and high grade and muscle-invasive tumors is depicted in Figure 6B. A logistic regression analysis applied to the significantly up-regulated genes identified a 2-gene signature (SOX2 and ALDH2) that discriminates (with an accuracy of 93%) muscle-invasive from non-muscle-invasive tumors. To confirm these findings, we also examined the protein expression levels of SOX2 and ALDH2 in paraffin-embedded tissue sections representative of the three histological subtypes of BC. Similarly to gene expression data, immunohistochemistry analysis revealed a higher fraction of cells with strong reactivity to SOX2 and ALDH2 in muscle-invasive tumors, in comparison with the non-muscle-invasive (Figure 6C). Despite the few number of samples analyzed our results show that muscle-invasive tumors possess a molecular signature assigned with a stem-like phenotype.

## DISCUSSION

A major issue in the management of BC patients is the identification of predictive markers for prognostic risk stratification. Although the majority of new BC cases are diagnosed as superficial papillary tumors, a high percentage of patients experienced disease relapse with an overall poor prognosis. So far, no molecularly targeted agents have been approved for the treatment of this disease, which underlies the need of novel targets for therapeutic intervention. In the present study we isolated and characterized CSCs from human cell lines and primary material and provided evidence that muscle-invasive BC harbor distinct cell subsets reflecting molecular features of stem-like cells and an aggressive phenotype characterized by enhanced chemoresistance and tumor initiating ability. Based on a real time PCR analysis we identified a potential molecular signature defined by SOX2 and ALDH2, validated at protein level in tumor tissue sections, that could be useful on the recognition of patients with muscle-invasive tumors that are more prone to experience disease progression or metastasis development.

We started by identifying stem-like cell populations within two invasive BC cell lines using a matrigel clonogenic assay and the Aldefluor surrogate assay as read-out strategies for CSCs enrichment. The former is based on the premise that only undifferentiated cells survive in serum-free medium growing in spherical colonies with all the other cell types dying by anoikis [28]; this approach is specifically useful to enrich the potential CSCs subpopulations when specific stem-like makers have not been defined. The second is based on the ALDH isozymes activity, which are members of the NAD(P)+ family and are involved in the detoxification of a wide variety of aldehydes, allowing the conversion of retinol to retinoic acid, a function that has been linked to stemness features of CSCs.

Our results demonstrated that B27-supplemented serum-free containing bFGF/EGF medium provides an optimal condition for isolation and enrichment of a cell subpopulation that fulfills the functional criteria of CSCs. The characterization of these cells showed a marked expression of SOX2, which together with POU5F1 (OCT4) and NANOG form a core transcriptional network governing self-renewal and maintenance of pluripotency of adult and embryonic stem cells [27, 29, 30]. The last two are equally expressed in parental cells and spheres suggesting that these factors are multifunctional and may have an oncogenic role, other than regulating self-renewal and differentiation. In agreement, Chang *et al*. detected high expression levels of OCT4 in BC specimens and found a correlation with tumor progression and metastasis through the activation of metalloproteinases regulators of cell motility [31].

Given the parallelism between CSCs and normal stem cells, it has been speculated that the regulatory networks that control stem cells are active in specific tumors cells. The expression of self-renewal transcription factors have been found preferentially expressed in high-grade (poorly differentiated) tumors of various types including glioblastomas and BC, while well-differentiated tumors displayed an opposite pattern [32–34]; these evidences suggest that the degree of tumor differentiation is determined, in part by the concerted activity of some of these key factors that ultimately contribute to a tumor aggressive behavior and to a poor prognosis.

Considering that BC arises from transformation in progenitor slow-cycling urothelial basal cells, attempts to isolate putative CSCs have been based on expression of surface markers restricted to basal cells that is thought to harbors multipotent stem cells required for normal tissue homeostasis. Our results showed a significant co-upregulation of CD44 and of basal-type KRT14, whose expression is restricted to undifferentiated basal cells within the normal urothelium. This cytokeratin is reported as a primitive stem cell marker precursor to KRT5 and KRT20 in urothelial differentiation and has been the basis for differentiation risk stratification of BC patients, being consistently associated with tumor recurrence and poor overall survival, independent of established and clinical pathological variables [35, 36]. We also observed a significant up-regulation of CD47 in the surface of spheres, which act as a “don't eat me” signal for phagocytic cells after interaction with receptor signal-regulatory protein alpha (SIRPα) on macrophages. This ligand has been found up-regulated in leukemic stem cells and more recently in a CD44^+^ subpopulation in invasive BC samples, suggesting that CSCs uses CD47 to escape immune system surveillance through evasion macrophage phagocytosis [37, 38]. In addition to this self-defense mechanism, we also observed a significant up-regulation of genes encoding P-glycoprotein and BCRP in sphere-forming cells enabling them to efflux harmful toxins, xenobiotics and chemotherapeutical drugs that are detrimental to cancer cells [39].

Furthermore, the analysis of ALDH isozymes showed a marked expression of ALDH1A1 and ALDH2 in spheres relatively to parental cells, but not of ALDH7A1, that overall contributes to ALDH activity measured by Aldefluor assay. Contrarily to some groups that found higher levels of ALDH7A1 isoform in prostate cancer bone metastasis, we found no correlation between bladder CSCs and this specific isoform, reflecting the high heterogeneity inherent to cancer type and tissue/cell of origin [40]. It is likely that the enzymatic activity of ALDH1A1 and ALDH2, together with the high levels of ABC-transporters, accounts to the highly chemoresistance profile exhibited by sphere-forming cells to MTX and CIS treatment, which demonstrated efficacy against parental cells.

We cannot exclude that other ALDH isoforms can contribute to the Aldefluor activity and chemoresistance profile observed in these cells. Indeed, ALDH1A1 along with ALDH2, ALDH1A3, ALDH6A1, ALDH7A1 or ALDH9A1 were found to be preferentially expressed in stem-like cell populations of breast and prostate cancer, but ALDH2 and ALDH7A1 were found to be part of an overlap in a gene expression profile analysis in different stem cell populations [26, 41, 42].

Our results clearly demonstrated the inefficacy of MTX and CIS against sphere-derived cells as indicated by the MTT and apoptosis assays, which is in agreement with previous studies in several other tumors, thus confirming the intrinsic chemoresistance of stem-like cells to conventional chemotherapeutic drugs, as well as to radiotherapy [39, 43], which are important cancer treatment strategies.

Previous studies showed that bladder CSCs isolated on the basis of the Aldefluor assay have enhanced resistance to CIS [44] providing evidence for a major role of ALDH1A1 isoform in conveying resistance to CIS in BC. We observed that 8–20% of the whole-cell population of each cell line is ALDH positive, which is in agreement with previous findings in BC cell lines and human tumor samples [24]. Additionally, we observed a substantial increase in the percentage of ALDH^+^ cells following short-term incubation with CIS or MTX. This later finding suggests that there is a phenotypic transition from a non-CSC to a CSC state (considering ALDH activity as a readout for stemness) when tumor cells experienced a cytotoxic environment. Other studies have also observed this switch through the interconversion of CD44^−^ in CD44^+^ prostate cancer cells and ABCG2^−^ in ABCG2^+^ breast cancer cell as a result of microenvironmental changes [45, 46]. These findings suggest that at least some cells within the tumor population harbor a certain degree of plasticity enable them to co-opt for a stemness phenotype dependent on microenvironmental signals. This plasticity in addition to the inherent heterogeneity of the carcinogenic process, suggests the co-existence of cancer cells with distinct functional and phenotypic stem-like properties within tumors. Accordingly, ALDH^+^ sorted cells showed a stemness signature that partially overlap with that of sphere-forming population of individual cell lines, suggesting that these functional assays might identify distinct cancer stem-like subsets differing in molecular characteristics. For instance, HT-1376 ALDH^+^ sorted cells overexpress the three-pluripotency transcription factors (POU5F1, NANOG and SOX2), while ALDH^+^ UM-UC3 cells only express POU5F1, differing both from sphere-derived cells that solely express SOX2. The expression of ALDH isozymes responsible for ALDH activity also differs between samples, being ALDH1A1 the only commonly expressed across different CSCs subsets. The two ABC transporters that were remarkably up regulated in spheres were not found in HT-1376 ALDH^+^ cells, which present high levels of the three ALDH isoforms analyzed. Also in the UM-UC3 cell line, it was observed a differential expression between spheres and ALDH^+^ cells, with the ALDH^+^ cells displaying high levels of ALDH1A1 and BCRP (ABCG2) while spheres showed a substantial higher expression of all drug-resistance related genes except ALDH7A1. In addition, like spheres, the ALDH^+^ fraction also shared some markers with urothelium basal cells; however, the hyaluronic acid CD44 that was up-regulated in spheres (at gene and protein level) versus parental cells was found ubiquitously expressed in the ALDH^+^ and ALDH^−^ subsets sorted from the UM-UC3 cell line, which reflects the functional ambiguity of this putative marker in CSCs maintenance. Similar stories hold true for CD133 in glioblastoma [47] and for CD44 and ALDH^+^ in breast cancer [22]. Even though the gene expression signature of sphere-forming cells did not entirely overlap with that of sorted ALDH^+^ cells, both cell populations express a set of stemness-related genes, which suggests the co-existence of CSCs pools regulated by distinct signaling pathways that are probably related with the diversity of oncogenic transformations and microenvironmental factors occurring in individual BC during tumor growth. Likewise, such heterogeneity was described by Boesch *et al*. in ovarian cancer cells. In this study, they identified multiple phenotypically distinct subpopulations of stem-like cells even within homogeneous cell line models and between cell lines, which reflects the high level of plasticity and likely influences in tumor progression and treatment response [48].

Taken together, these results suggest that not all putative CSCs markers are applicable to identify CSCs in tumor samples; in addition, a single marker or functional property may not always be effective to define a stem-like population, generating conflicting results. It is therefore conceivable that a combination of markers should be used to more narrowly refine the CSC phenotype in BC as well as in other tumors.

The ultimate hallmark of CSCs is their ability to initiate and support tumor growth *in vivo* in immunocompromised mice. We observed that both parental and sphere-derived cells form tumors when inoculated subcutaneously in mice; however, spheres were able to grow faster and developed larger tumors than their corresponding parental cells, clearly demonstrating their enhanced tumorigenic potential. Additionally, we demonstrated that CSCs, when engrafted orthotopically in the bladder, developed a phenotypically heterogeneous tumor resembling the clinical and histological features of primary invasive BC; under these conditions, a minority of cells expressing markers of undifferentiated cells including SOX2, ALDH2 and CD47, was observed, together with an expanded population of proliferating differentiated cells. These results suggest that sphere-derived cells in an organ-specific microenvironment are able to self-renew and to generate the downstream differentiated tumor cells, fulfilling the functional criteria for CSCs. Likewise in the subcutaneous model, parental cells also formed an orthotopic tumor resembling a primary BC (see Supplementary Figure S2), but it required the inoculation of a 4-fold more cells because of the lower frequency of tumor-initiating cells in the bulk tumor cell population.

Finally, we extended our study to a cohort of human tumor samples collected during endoscopic resection to evaluate the pathological significance of the analyzed stemness-related genes and investigate whether these markers can provide additional prognostic information for both staging and grading. Despite the small number of samples, we found a significant up-regulation of some stemness-related markers, (SOX2 and ALDH isozymes ALDH1A1 and ALDH2) in muscle-invasive tumors versus non-muscle-invasive. Additionally, a logistic regression analysis identified a 2-gene signature (SOX2 and ALDH2) that discriminates (with an accuracy of 93%) muscle-invasive from non-muscle-invasive tumors. The oncogenic role of SOX2 has been described in stem-like cells of various cancers. Boumahdi *et al*. showed that SOX2 is up regulated in CSCs of squamous skin tumors and regulates the expression of key genes controlling tumor stemness, survival, proliferation, adhesion, invasion and the paraneoplastic syndrome [49]. Also, Nakatsugawa *et al*. demonstrated the role of SOX2 in the maintenance of stemness and tumorigenicity of human lung adenocarcinoma cells. They found the down-regulation of SOX2 by siRNA abrogated completely the tumorigenicity further suggesting this transcription factor regulates the expression of key genes and pathways that control tumor initiation and progression [50].

Since patients with muscle-invasive tumors present substantial risk of disease progression and recurrence, this signature could be of predictive value as it may prospectively identify BC patients that would particularly benefit from a more aggressive therapeutic intervention targeting CSCs at earlier time points. Obviously, before its use in clinical practice, this gene profile must be validated using a larger number of BC samples, which will be done in the forthcoming phases of this research.

In conclusion, we provided evidence that BC contains distinct cell populations exhibiting a differential gene expression pattern strongly related with stem cell pluripotency, basal urothelial stem cells or drug-detoxifying systems that are highly resistant to chemotherapy and endowed with tumor initiating ability. In addition, by employing distinct functional methods, we were able to shown that individual tumors harbor distinct cell populations expressing different CSCs markers. The preferential expression of at least two stemness-related markers in muscle-invasive tumors strongly reinforces the role of CSCs as a driving force in the pathogenesis and relapse of invasive BC, highlighting the need to delineate novel therapeutic approaches considering CSCs as a target population.

## MATERIALS AND METHODS

### Cell culture and matrigel clonogenic assay

Human HT-1376 and UM-UC3 BC cell lines derived from high grade transitional cell carcinoma (ATCC, Manassas, VA, USA) were cultured in RPMI 1640 medium (Gibco, Scotland, UK) supplemented with 10% heat inactivated fetal bovine serum (FBS) (Gibco, Carlsbad, CA), 200 mM of L-glutamine (Sigma) and penicillin-streptomycin (Gibco, penicillin: 100 IU/mL and streptomycin: 100 mg/mL) at 37°C in 5% CO_2_.

The matrigel clonogenic assay was performed as described elsewhere [28] with minor modifications. Briefly, 40 μL of single cell suspension (2 × 10^5^/mL) was added to 60 μL of cold Matrigel (BD Biosciences) and plated around the rims of wells in a 12-well plate, and allowed to solidify at 37°C. After 30 min, 1 mL of serum-free DMEM/F12 medium (Sigma) supplemented with 1% penicillin/streptomycin (Gibco), 20 nM progesterone (Sigma), 100 μM putrescine (Sigma), 1% insulin-transferrin-selenium A supplement (Gibco), 10 ng/ml basic fibroblast growth factor (bFGF, Peprotech EC, London, UK), 10 ng/mL human recombinant epidermal growth factor (EGF, Sigma) and 2% of B27 supplement (Gibco). The medium was replenished every 3 days. Ten days after plating, the medium was removed and Matrigel was digested with 500 μL of dispase solution (Invitrogen) 1 mg/mL, dissolved in serum-free DMEM/F12 medium for 30 min at 37°C. Digested cultures were pelleted and incubated with 400 μL of accutase solution (Gibco) for 5 minutes. Dissociated cells were maintained in the sphere-forming medium in the 37°C incubator for 30 min prior to use.

### Aldefluor assay and fluorescence-activated cell sorting (FACS)

Both parental and corresponding sphere-forming cells were assayed for aldehyde dehydrogenase (ALDH) activity using the Aldefluor kit (Stem Cell Technologies) according to the manufacturer's instructions. FACS was performed on a BD FACSAria III (BD Biosciences) cell-sorting system. Data was analyzed with the FlowJo software (Tree Star, Inc, Ashland, USA).

To further verify whether treatment with chemotherapeutic agents might induce an enrichment of ALDH-positive cells, we performed an ALDH enzymatic assay in parental cells following 24 h incubation with 10 μM CIS (Teva Pharma, Portugal) or 10 μM MTX (Teva Pharma, Portugal).

### Gene expression by quantitative real-time PCR analysis (qRT-PCR)

Total RNA from adherent parental cell lines and their respective sphere-forming cells was extracted using TRIzol (Gibco). Total RNA from ALDH^+^ and ALDH^−^ sorted cells was extracted using the ReliaPrep RNA Cell Miniprep System (Promega) following the manufacturer's protocol. The quantity and quality of isolated RNA was measured by the ND-1000 spectrophotometer (NanoDrop Technologies). Reverse transcription from 1 μg of total RNA was performed using NZY First-Strand cDNA Synthesis kit (Nzytech) in a reaction volume of 20 μl. Quantitative real-time PCR analysis (qRT-PCR) was performed using a Quantitect SYBR Green qRT-PCR kit (Biorad) for NANOG, POU5F1 (OCT4), SOX2, ABCG2 (BCRP), ABCB1 (PGP), ALDH1A1, ALDH7A1 and ALDH2 in a Bio-Rad CFX 96 Thermal Cycler (Bio-Rad Laboratories, CA, United States).

qRT-PCR amplification of CD47, CD44 and KRT14 genes was performed using the SYBR-Green I Master (Roche) according to the manufacturer's instructions in a LightCycler 480 II system (Roche Diagnostics, Mannheim, Germany). mRNA expression was normalized to three housekeeping genes: 18S, GAPDH and HRPT-1 using the δδCt method and Bio-Rad CFX Manager™ 3.0 software. Primers sequences are listed on Supplementary Table S1.

### Immunofluorescence and laser confocal microscopy

Cytospins were prepared with freshly dissociated cells. After spinning, cells were fixed with 4% paraformaldehyde for 10 min and permeabilized with 1% Triton X-100 in PBS for 10 min. Cells were blocked with 10% of normal goat serum for 30 min at 37°C, and then incubated with a primary antibody against CD44 (1:100; Santa Cruz Biotechnology), CD47 (1:100, Abcam) and KRT14 (1:100, Santa Cruz Biotechnology) for 1 h in humid atmosphere. After rising, cells were incubated with secondary goat anti-mouse IgG-Alexa Fluor 594-conjugated (1:100, Invitrogen), and the nuclei were counterstained with Hoechst (5 μg/mL). Negative controls were prepared by omitting staining with the primary antibody. Images were acquired in a confocal microscopy Zeiss LSM710 system (Carl Zeiss AG) using *a* × 63 1.4 NA oil immersion lens. The quantification of fluorescence intensities was performed using the NIH ImageJ 1.47v analysis software. Regions were drawn around each fluorescent cells and in a region without fluorescent objects for background subtraction. The corrected total cell fluorescence (CTCF) was determined using the following formula: CTCF = integrated intensity - (area for the selected cell × mean background). Data is represented as the mean of fluorescence intensity (MFI).

### Chemosensitivity assays

Parental monolayer cells and matched spheres were assayed for chemosensitivity to Cisplatin (CIS, Teva Pharma, Portugal) and methotrexate (MTX, Teva Pharma, Portugal) using the MTT colorimetric viability assay. Being CIS a cytotoxic drug-induced cell dead, we also evaluated cells’ susceptibility to apoptosis using Annexin V staining and caspase 3/7 activity.

### MTT viability assay

Cells were plated in 96-well plates and incubated with increasing concentrations of CIS (0.1 – 100 μM) or MTX (0.1 – 10 μM) during 48 h. After treatments, the medium was removed and 0.5 mg/mL MTT [3-(4,5-dimethylthiazol-2-yl)-2,5-diphenyltetrazolium bromide] (Sigma) was added to each well, for formation of formazan crystals. The precipitated dye was dissolved in 0.04M HCl (in isopropanol) and quantified at a wavelength of 570 nm, using 620 nm filter as a reference, in a microplate reader (Synergy^™^ HT, Biotek Instruments). Cell viability was expressed as the percentage of absorbance values of the treated wells related to the untreated control wells considered as 100%. Drug concentration required to inhibit cell viability by 50% (IC_50_) was estimated using a sigmoidal dose-response fitting using the OriginPro8 (OriginLab Corporation).

### Annexin V apoptosis detection by flow cytometry

Cells were incubated with 10 μM CIS for 48 h, which correspond to mean IC_50_ value of parental cells provided by the MTT assay. After treatments, cells were incubated with FITC-Annexin V and propidium iodide (PI) from Immunostep (Salamanca, Spain) following the manufacturer instructions. Stained cells were subjected to flow cytometry analysis using FACSCanto flow cytometer (BD Biosciences).

### Measurement of caspase 3/7 activity

Caspase 3/7 activity were measured in cells treated with 10 μM CIS for 48 h using the Caspase-Glo 3/7 assay Kit (Promega, Corp., Madison, WI) following manufacturer's instructions. The generated bioluminescent signal was measured in a microplate reader (Synergy^™^ HT, Biotek Instruments). Signal intensity of treated cells was normalized to corresponding untreated controls.

### Lentiviral transduction of cell lines

For luciferase (Luc) gene transduction, HT-1376 and UM-UC3 cells were incubated with a 1:3 mixture of lentiviral supernatants pLenti6/CMV/fLuc (Invitrogen) in RPMI-1640 medium containing polybrene (10 μg/mL, Millipore). After 48 h, fresh culture medium containing 8 μg/mL blasticidin S hydrocloride (Life Technologies, Basel, Switzerland) was added for selection of transduced cells. Clones with the highest bioluminescent signal were expanded for further studies. CSCs were isolated from Luc-transduced parental cells using the matrigel clonogenic assay as previously described.

### Animal studies

Animal studies were approved by the Institutional Ethics Committee of the Faculty of Medicine, University of Coimbra (ORBEA_91_2015/08) and were performed according to the local and international guidelines on animal experimentation. Female nude mice (Swiss nu/nu), 6–8 weeks old, were obtained from Charles River Laboratories, (Barcelona, Spain) and housed under pathogen free conditions in individual ventilated cages.

### Subcutaneous xenografts tumors

Animals were inoculated subcutaneously into the lower flank with 1×10^4^ of parental or corresponding sphere-forming cells resuspended in 100 μL of a 1:1 PBS/Matrigel mixture. Tumor growth was monitored weekly by whole-body bioluminescence imaging (BLI) on an IVIS Lumina XR (Caliper Life-Sciences, Hopkinton, MA, USA) following intraperitoneal administration of 150 mg/kg D-luciferin (Synchem, BHg, Germany) with the animals previously anesthetized with 100 mg/Kg ketamine and 2.5% of chlorpromazine solution. Bioluminescent images were analyzed using the Living Image software version 4.10 (Xenogen). A region of interest (ROI) was drawn around the tumor for quantification of the bioluminescent signal. Values are expressed photons/sec/cm^2^/sr. After 5 weeks, the animals were sacrificed by cervical dislocation and primary tumors were collected for histopathological analysis.

### Orthotopic bladder cancer model

The orthotopic implantation was performed as described by Chan *et al.* [51]. A 24-gauge catheter lubricated with xylocaine gel was inserted transurethrally into the bladder of anesthetized mice. After flushing with sterile PBS, the bladder was pre-treated with 0.1 mg/mL poly-L-lysine (Sigma, the Netherlands) for 30 min and then infused with a single-cell suspension containing 1×10^6^ of CSCs or 4×10^6^ of parental cells. The animals were kept under anesthesia with hind limbs up for 3 h before the catheter was removed to assure that the cells were retained in the bladder. Tumor growth was monitored weekly for 5 weeks by BLI as previously described above. The animals were sacrificed when presenting hematuria or when they lose 20% of initial body weight.

### Histological analysis

Tumors were fixed in formaldehyde 4% and processed for paraffin embedding. Sections of 3-μm were stained with hematoxylin and eosin (H&E), periodic acid-Schiff and Masson trichrome. Histological examination was done by two independent pathologists.

### Immunohistochemistry

Formalin-fixed paraffin-embedded tissue blocks were sectioned at 3-μm thickness and incubated in a Bench Mark Ultra Ventana, with primary antibodies against Ki67, clone SP6 (1:50, NeoMarkers, LabVision, Fremont, CA, USA), SOX2 clone D6D9 (1:100, Cell Signaling), CD44 clone DF1485 (1:50, Santa Cruz), CD47 clone B6M12.2 (1:100, Abcam) and ALDH2 clone EPR4493 (1:100, Abcam), for 30 min at 37°C. A 2-step multimer conjugated with peroxidase (UltraView Universal DAB Detection Kit, Ventana Medical Systems, Inc, Tucson, Arizona, USA) was applied for 8 min at 37°C, and reaction signal was developed with 3-3′-diamino benzidine tetrahydrochloride chromogen. Standard procedures were used for visualization and the intensity and percentage of positive staining was registered. Two investigators blinded to the data reviewed all slides independently.

### Clinical samples

Bladder tumor samples were obtained from 17 patients (14 males and 3 females) by transurethral resection, at Coimbra's University Hospital. The patients’ mean age was 74 years (ranging, 63–90) and all bladder samples were obtained after appropriate informed consent and ethical regulatory approval. A pathologist classified tumors stage and grade according to the WHO criteria (2004). At initial diagnosis all patients with primary diagnosis of BC were eligible for our study, and were submitted to a clinical history and a cystoscopy. Patients were submitted to an initial transurethral resection of the BC, according to the EAU Guidelines. Patients with superficial tumors were treated with intravesical instillation of Mitomycin C while radical cystectomy was applied to patients with invasive tumors. The excluding criteria were: recurrent BC, previous pelvic radiotherapy, intravesical or systemic chemotherapy, Karnofsky performance status < 90, concomitant bladder diseases (tuberculosis, schistosomiasis, cystitis) and significant comorbidities with short survival expectancy. A minimal follow up of one year was considered.

After transurethral resection, a tumour sample was immersion-fixed in 4% buffered formaldehyde and processed for paraffin sectioning to histological and immunohistochemistry (IHC) analysis. A small section of tissue sample was also immediately submerged in RNA*later* solution (QIAGEN, Netherlands) for RNA assays. Total RNA extraction from tumor samples was performed using MagNA Lyser Green Beads (Roche Diagnostics, Mannheim, Germany) and High Pure RNA Isolation Kit, Tissue (Roche Diagnostics, Mannheim, Germany), according to the manufacturer's instructions. Subsequent cDNA synthesis and qRT-PCR were performed as described above. As a control, bladder samples were taken from patients who had bladder transurethral surgery for non-bladder cancer pathologies.

### Statistical analyses

Data are reported as the means + SEM of the indicated number of experiments. Statistical analyses were performed using the Student's *t*-test for two samples and ANOVA with Bonferroni correction for multiple comparisons. Statistical significance was defined as: *p < 0.05, **p < 0.01 and ***p < 0.001.

A logistic regression model was carried out aiming at discriminate non-muscle-invasive from muscle-invasive tumors. The up-regulated genes were used as independent variables in a backward (conditional) procedure. The Wald test was considered to assess the statistical meaning of the variables. The performance of the regression was evaluated by ROC analysis and the area under the curve (AUC) was determined.

Statistical analysis and graphic illustrations were performed using GraphPad Prism 6.0 software (San Diego, CA).

## SUPPLEMENTARY FIGURES AND TABLE



## References

[1] Burger M, Catto JW, Dalbagni G, Grossman HB, Herr H, Karakiewicz P, Kassouf W, Kiemeney LA, La Vecchia C, Shariat S, Lotan Y (2013). Epidemiology and risk factors of urothelial bladder cancer. European urology.

[2] Ye F, Wang L, Castillo-Martin M, McBride R, Galsky MD, Zhu J, Boffetta P, Zhang DY, Cordon-Cardo C (2014). Biomarkers for bladder cancer management: present and future. American journal of clinical and experimental urology.

[3] Ploeg M, Aben KK, Kiemeney LA (2009). The present and future burden of urinary bladder cancer in the world. World journal of urology.

[4] Cheung G, Sahai A, Billia M, Dasgupta P, Khan MS (2013). Recent advances in the diagnosis and treatment of bladder cancer. BMC medicine.

[5] Anastasiadis A, de Reijke TM (2012). Best practice in the treatment of nonmuscle invasive bladder cancer. Therapeutic advances in urology.

[6] Dancik GM, Owens CR, Iczkowski KA, Theodorescu D (2014). A cell of origin gene signature indicates human bladder cancer has distinct cellular progenitors. Stem cells.

[7] Celia-Terrassa T, Meca-Cortes O, Mateo F, de Paz AM, Rubio N, Arnal-Estape A, Ell BJ, Bermudo R, Diaz A, Guerra-Rebollo M, Lozano JJ, Estaras C, Ulloa C, Alvarez-Simon D, Mila J, Vilella R (2012). Epithelial-mesenchymal transition can suppress major attributes of human epithelial tumor-initiating cells. The Journal of clinical investigation.

[8] Chan KS, Volkmer JP, Weissman I (2010). Cancer stem cells in bladder cancer: a revisited and evolving concept. Current opinion in urology.

[9] van der Horst G, Bos L, van der Pluijm G (2012). Epithelial plasticity, cancer stem cells, and the tumor-supportive stroma in bladder carcinoma. Molecular cancer research : MCR.

[10] Hepburn AC, Veeratterapillay R, Williamson SC, El-Sherif A, Sahay N, Thomas HD, Mantilla A, Pickard RS, Robson CN, Heer R (2012). Side population in human non-muscle invasive bladder cancer enriches for cancer stem cells that are maintained by MAPK signalling. PloS one.

[11] Visvader JE, Lindeman GJ (2012). Cancer stem cells: current status and evolving complexities. Cell stem cell.

[12] Ho PL, Kurtova A, Chan KS (2012). Normal and neoplastic urothelial stem cells: getting to the root of the problem. Nature reviews Urology.

[13] Visvader JE, Lindeman GJ (2008). Cancer stem cells in solid tumours: accumulating evidence and unresolved questions. Nature reviews Cancer.

[14] Hatina J, Schulz WA (2012). Stem cells in the biology of normal urothelium and urothelial carcinoma. Neoplasma.

[15] Goncalves C, Martins-Neves SR, Paiva-Oliveira D, Oliveira VE, Fontes-Ribeiro C, Gomes CM (2015). Sensitizing osteosarcoma stem cells to doxorubicin-induced apoptosis through retention of doxorubicin and modulation of apoptotic-related proteins. Life sciences.

[16] Alisi A, Cho WC, Locatelli F, Fruci D (2013). Multidrug resistance and cancer stem cells in neuroblastoma and hepatoblastoma. International journal of molecular sciences.

[17] Perona R, Lopez-Ayllon BD, de Castro Carpeno J, Belda-Iniesta C (2011). A role for cancer stem cells in drug resistance and metastasis in non-small-cell lung cancer. Clinical & translational oncology : official publication of the Federation of Spanish Oncology Societies and of the National Cancer Institute of Mexico.

[18] Dotsikas G, Konowalchuk T, Major PP, Kovac PE, Ward GK, Stewart SS, Price GB, Elhilali MM, Mackillop WJ (1987). Cellular heterogeneity in normal and neoplastic human urothelium: a study using murine monoclonal antibodies. British journal of cancer.

[19] Bentivegna A, Conconi D, Panzeri E, Sala E, Bovo G, Vigano P, Brunelli S, Bossi M, Tredici G, Strada G, Dalpra L (2010). Biological heterogeneity of putative bladder cancer stem-like cell populations from human bladder transitional cell carcinoma samples. Cancer science.

[20] Yu C, Yao Z, Dai J, Zhang H, Escara-Wilke J, Zhang X, Keller ET (2011). ALDH activity indicates increased tumorigenic cells, but not cancer stem cells, in prostate cancer cell lines. In vivo.

[21] Kuncova J, Kostrouch Z, Viale M, Revoltella R, Mandys V (2005). Expression of CD44v6 correlates with cell proliferation and cellular atypia in urothelial carcinoma cell lines 5637 and HT1197. Folia biologica.

[22] Ginestier C, Hur MH, Charafe-Jauffret E, Monville F, Dutcher J, Brown M, Jacquemier J, Viens P, Kleer CG, Liu S, Schott A, Hayes D, Birnbaum D, Wicha MS, Dontu G (2007). ALDH is a marker of normal and malignant human mammary stem cells and a predictor of poor clinical outcome. Cell stem cell.

[23] Wu A, Luo W, Zhang Q, Yang Z, Zhang G, Li S, Yao K (2013). Aldehyde dehydrogenase 1, a functional marker for identifying cancer stem cells in human nasopharyngeal carcinoma. Cancer letters.

[24] Su Y, Qiu Q, Zhang X, Jiang Z, Leng Q, Liu Z, Stass SA, Jiang F (2010). Aldehyde dehydrogenase 1 A1-positive cell population is enriched in tumor-initiating cells and associated with progression of bladder cancer. Cancer epidemiology, biomarkers & prevention : a publication of the American Association for Cancer Research, cosponsored by the American Society of Preventive Oncology.

[25] Marcato P, Dean CA, Pan D, Araslanova R, Gillis M, Joshi M, Helyer L, Pan L, Leidal A, Gujar S, Giacomantonio CA, Lee PW (2011). Aldehyde dehydrogenase activity of breast cancer stem cells is primarily due to isoform ALDH1A3 and its expression is predictive of metastasis. Stem cells.

[26] Marcato P, Dean CA, Giacomantonio CA, Lee PW (2011). Aldehyde dehydrogenase: its role as a cancer stem cell marker comes down to the specific isoform. Cell cycle.

[27] Amini S, Fathi F, Mobalegi J, Sofimajidpour H, Ghadimi T (2014). The expressions of stem cell markers: Oct4, Nanog, Sox2, nucleostemin, Bmi, Zfx, Tcl1, Tbx3, Dppa4, and Esrrb in bladder, colon, and prostate cancer, and certain cancer cell lines. Anatomy & cell biology.

[28] Xin L, Lukacs RU, Lawson DA, Cheng D, Witte ON (2007). Self-renewal and multilineage differentiation *in vitro* from murine prostate stem cells. Stem cells.

[29] Jozwicki W, Brozyna AA, Siekiera J (2014). Expression of OCT4A: the first step to the next stage of urothelial bladder cancer progression. International journal of molecular sciences.

[30] Dick JE (2008). Stem cell concepts renew cancer research. Blood.

[31] Chang CC, Shieh GS, Wu P, Lin CC, Shiau AL, Wu CL (2008). Oct-3/4 expression reflects tumor progression and regulates motility of bladder cancer cells. Cancer research.

[32] Sanchez-Carbayo M, Socci ND, Lozano J, Saint F, Cordon-Cardo C (2006). Defining molecular profiles of poor outcome in patients with invasive bladder cancer using oligonucleotide microarrays. Journal of clinical oncology : official journal of the American Society of Clinical Oncology.

[33] Sun L, Hui AM, Su Q, Vortmeyer A, Kotliarov Y, Pastorino S, Passaniti A, Menon J, Walling J, Bailey R, Rosenblum M, Mikkelsen T, Fine HA (2006). Neuronal and glioma-derived stem cell factor induces angiogenesis within the brain. Cancer cell.

[34] Ben-Porath I, Thomson MW, Carey VJ, Ge R, Bell GW, Regev A, Weinberg RA (2008). An embryonic stem cell-like gene expression signature in poorly differentiated aggressive human tumors. Nature genetics.

[35] Ho PL, Lay EJ, Jian W, Parra D, Chan KS (2012). Stat3 activation in urothelial stem cells leads to direct progression to invasive bladder cancer. Cancer research.

[36] Volkmer JP, Sahoo D, Chin RK, Ho PL, Tang C, Kurtova AV, Willingham SB, Pazhanisamy SK, Contreras-Trujillo H, Storm TA, Lotan Y, Beck AH, Chung BI, Alizadeh AA, Godoy G, Lerner SP (2012). Three differentiation states risk-stratify bladder cancer into distinct subtypes. Proceedings of the National Academy of Sciences of the United States of America.

[37] Chan KS, Espinosa I, Chao M, Wong D, Ailles L, Diehn M, Gill H, Presti J, Chang HY, van de Rijn M, Shortliffe L, Weissman IL (2009). Identification, molecular characterization, clinical prognosis, and therapeutic targeting of human bladder tumor-initiating cells. Proceedings of the National Academy of Sciences of the United States of America.

[38] Majeti R, Chao MP, Alizadeh AA, Pang WW, Jaiswal S, Gibbs KD, van Rooijen N, Weissman IL (2009). CD47 is an adverse prognostic factor and therapeutic antibody target on human acute myeloid leukemia stem cells. Cell.

[39] Abdullah LN, Chow EK (2013). Mechanisms of chemoresistance in cancer stem cells. Clinical and translational medicine.

[40] van den Hoogen C, van der Horst G, Cheung H, Buijs JT, Pelger RC, van der Pluijm G (2011). The aldehyde dehydrogenase enzyme 7A1 is functionally involved in prostate cancer bone metastasis. Clinical & experimental metastasis.

[41] Moreb JS (2008). Aldehyde dehydrogenase as a marker for stem cells. Current stem cell research & therapy.

[42] Blanpain C, Lowry WE, Geoghegan A, Polak L, Fuchs E (2004). Self-renewal, multipotency, and the existence of two cell populations within an epithelial stem cell niche. Cell.

[43] Martins-Neves SR, Lopes AO, do Carmo A, Paiva AA, Simoes PC, Abrunhosa AJ, Gomes CM (2012). Therapeutic implications of an enriched cancer stem-like cell population in a human osteosarcoma cell line. BMC cancer.

[44] Falso MJ, Buchholz BA, White RW (2012). Stem-like cells in bladder cancer cell lines with differential sensitivity to cisplatin. Anticancer research.

[45] Patrawala L, Calhoun T, Schneider-Broussard R, Zhou J, Claypool K, Tang DG (2005). Side population is enriched in tumorigenic, stem-like cancer cells, whereas ABCG2+ and ABCG2- cancer cells are similarly tumorigenic. Cancer research.

[46] Patrawala L, Calhoun T, Schneider-Broussard R, Li H, Bhatia B, Tang S, Reilly JG, Chandra D, Zhou J, Claypool K, Coghlan L, Tang DG (2006). Highly purified CD44+ prostate cancer cells from xenograft human tumors are enriched in tumorigenic and metastatic progenitor cells. Oncogene.

[47] Chen R, Nishimura MC, Bumbaca SM, Kharbanda S, Forrest WF, Kasman IM, Greve JM, Soriano RH, Gilmour LL, Rivers CS, Modrusan Z, Nacu S, Guerrero S, Edgar KA, Wallin JJ, Lamszus K (2010). A hierarchy of self-renewing tumor-initiating cell types in glioblastoma. Cancer cell.

[48] Boesch M, Zeimet AG, Reimer D, Schmidt S, Gastl G, Parson W, Spoeck F, Hatina J, Wolf D, Sopper S (2014). The side population of ovarian cancer cells defines a heterogeneous compartment exhibiting stem cell characteristics. Oncotarget.

[49] Boumahdi S, Driessens G, Lapouge G, Rorive S, Nassar D, Le Mercier M, Delatte B, Caauwe A, Lenglez S, Nkusi E, Brohee S, Salmon I, Dubois C, del Marmol V, Fuks F, Beck B (2014). SOX2 controls tumour initiation and cancer stem-cell functions in squamous-cell carcinoma. Nature.

[50] Nakatsugawa M, Takahashi A, Hirohashi Y, Torigoe T, Inoda S, Murase M, Asanuma H, Tamura Y, Morita R, Michifuri Y, Kondo T, Hasegawa T, Takahashi H, Sato N (2011). SOX2 is overexpressed in stem-like cells of human lung adenocarcinoma and augments the tumorigenicity. Laboratory investigation; a journal of technical methods and pathology.

[51] Chan E, Patel A, Heston W, Larchian W (2009). Mouse orthotopic models for bladder cancer research. BJU international.

