# Supplementary material for: Functional and molecular characterization of cancer stem-like cells in bladder cancer: a potential signature for muscle-invasive tumors
Source: Oncotarget. 2015 Oct 5;6(34):36185–201. doi: 10.18632/oncotarget.5517 (PMC4742170; doi:10.18632/oncotarget.5517)
Supplement: Supplementary file 1 [file oncotarget-06-36185-s001.pdf]

SUPPLEMENTARY FIGURES AND TABLE

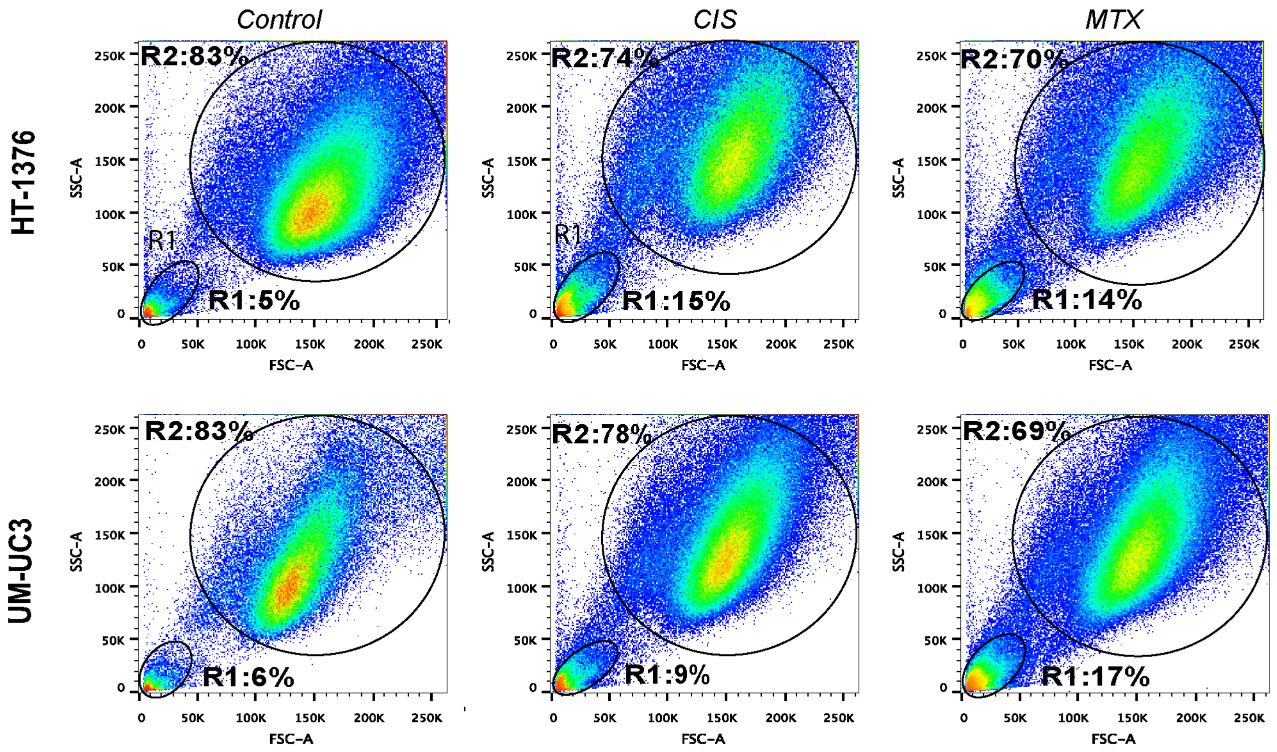

Supplementary Figure S1: Short-term chemotherapy has minimal effects on tumor cells viability. Representative flow cytometry dot plots of BC cells upon short-term single exposure to chemotherapy. Cells were treated with 10 µM CIS or 10 µM MTX for 24 hours and then subjected to flow cytometry analysis. R1 represents the percentage of dead cells and R2 the percentage of viable cells of the whole-cell population.

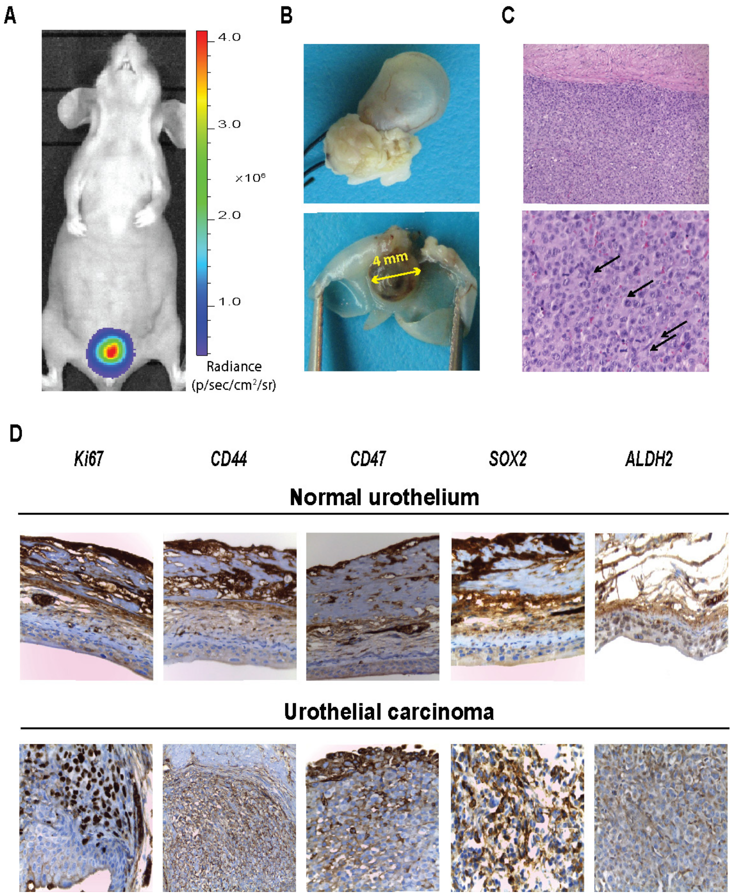

Supplementary Figure S2: Bladder cancer induced by orthotopic inoculation of adherent HT-1376 cells. A. Representative BLI image of a mouse that was inoculated intravesically with 4×10^6 adherent HT-1376 cells at 5 weeks. **B.** Macroscopic examination of a papillary tumor protruding into the bladder lumen on day 40. **C.** Representative H&E–stained (×200) and high mitotic count (×400). **D.** Immunohistochemical staining for Ki67, CD44, CD47, SOX2 and ALDH2 in serial sections of normal urothelium (upper row) and in the orthotopic tumor (lower row). Original magnification: ×400.

Supplementary Table S1: Primer sequences used in real-time RT-qPCR analysis

Gene	Primer	Primer Sequence/ENSEMBL Gene ID
ALDH1A1	Forward	5′-TGGCTTATCAGCAGGAGTGT-3′
ALDH1A1	Reverse	5′-GCAATTCACCCACACTGTTC-3′
ALDH2	Forward	5′-CGAGGTCTTCTGCAACCAG-3′
ALDH2	Reverse	5′-GCCTTGTCCACATCTTCCTT-3′
ALDH7A1	Forward	5′-CAACGAGCCAATAGCAAGAG-3′
ALDH7A1	Reverse	5′-GCATCGCCAATCTGTCTTAC-3′
SOX2	Forward	5′-CATGCACCGCTACGACG-3′
SOX2	Reverse	5′-CGGACTTGACCACCGAAC-3′
NANOG	Forward	5′-GATGCCTCACACGGAGACTG-3′
NANOG	Reverse	5′-GCAGAAGTGGGTTGTTTGCC-3′
POU5F1	Forward	5′-TCTGCATCCCTTGGATGTGC-3′
POU5F1	Reverse	5′-GTGTGGCCCCAAGGAATAGT-3′
ABCB1	Forward	5′-GAGGAAGACATCACCAGGTATGC-3′
ABCB1	Reverse	5′-GCCAGGCACCAAAATGAAACC-3′
ABCG2	Forward	5′-TCAGCGGATACTACAGAGTGTCATC-3′
ABCG2	Reverse	5′-ATCTGCCTTTGGCTTCAATCCTAA-3′
HPRT-1	Forward	5′-TGACACTGGCAAAACAATG-3′
HPRT-1	Reverse	5′-GGCTTATATCCAACACTTCG-3′
18S	Forward	5′-GAAGATATGCTCATGTGGTGTTG-3′
18S	Reverse	5′-CTTGTACTGGCGTGGATTCTG-3′
GAPDH	Forward	5′-ACAGTCAGCCGCATCTTC-3′
GAPDH	Reverse	5′-GCCCAATACGACCAAATCC-3′
CD44		ENST00000278385
CD47		ENST00000398257
KRT14		ENST00000167586

All Cycle threshold (Ct) values of the analyzed genes were normalized to the housekeeping genes, *i.e.*, HRPT-1, 18S and GAPDH.
